# Extensions of the Galperin Transformation Matrices for Triaxial Seismometers

**DOI:** 10.3390/s23010026

**Published:** 2022-12-20

**Authors:** Talso C. P. Chui, Andrew Erwin, Inseob Hahn

**Affiliations:** Jet Propulsion Laboratory, California Institute of Technology, Pasadena, CA 91109, USA

**Keywords:** Galperin, seismometer, Brownian noise, distributed masses, temperature sensitivity

## Abstract

Since its invention in 1955, the Galperin symmetric triaxial seismometer has been widely used for seismic detection on Earth, and most recently on the planet Mars. In this paper, we present detailed physics of such seismometers, which has not yet been published in open literature. We extended Galperin’s original work, which is based on idealized geometry and assumptions, to include more practical cases, including (1) non-idealized tilt angles of its component seismometers; (2) component seismometers that are not exactly oriented 120° apart; (3) distributed mass on the boom; and (4) the case of operations at lower frequencies.

## 1. Introduction

A seismometer is a precision instrument that measures ground motion through the principle of inertia by suspending a mass from an elastic element [1]. While rotational ground motion can be measured [2], traditionally seismic detection has focused on the translational motion [3]. Earlier seismometers measured translational ground motion in the cardinal X, Y, and Z coordinate frame, corresponding to east/west, north/south, and vertical ground motion [4,5].

**Figure 1:**
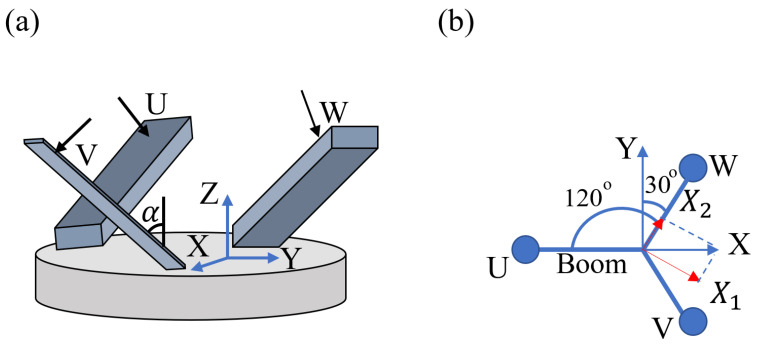
(**a**) The configuration of the triaxial seismometer used in the derivation. The UVW vectors are normal to the boom planes. The UVW axes are orthogonal only when the angle *α* is at the Galperin tilt angle, 35.26 deg. (**b**) The simplified top view of the UVW seismometer.

In the symmetric triaxial seismometer, three single-axis sensors are spaced equally apart on a circle in the horizontal plane while the vertical component of the sensors all experience the same gravitational acceleration. The UVW sensing directions are given by the direction orthogonal to the boom, which suspends the moving mass. As shown in Figure 1, the tilt angle α is the angle that the boom makes with respect to the vertical direction (it is also the same angle that the sensing axis makes with the horizontal axis). For the Galperin symmetric triaxial seismometer, α is $\tan^{-1}\frac{1}{\sqrt{2}}$ or 35.26° and the UVW axes create an orthogonal reference frame [6,7,8,9,10].

State-of-the-art seismometers, such as the Streckeisen STS-2 and the Nanometrics Inc. Trillium Compact, employ a symmetric Galperin configuration [11,12,13,14]. Recently, a microseismometer with a Galperin configuration has been proposed for Lunar deployment [15]. Despite the ubiquity of the Galperin configuration, there are some advantages to the traditional cardinal X, Y, and Z configurations. The Streckeisen STS-1 seismometer, which is one of the most sensitive seismometers deployed on Earth [16], employs the cardinal configuration [5]. A new optical-based seismometer is in development and will use the cardinal configuration [17]. A disadvantage of the cardinal configuration is that the vertical sensor must be designed separately from the horizontal sensors as it experiences an additional force from the local gravity. In contrast, the symmetric triaxial seismometer is created with three (ideally) identical sensors, providing manufacturing, calibration, and control benefits [9].

While the Galperin configuration has primarily been used for triaxial symmetric seismometers, the Insight Seismic Experiment for Interior Structure Very Broad Band (SEIS-VBB) seismometer deployed on Mars [18] uses a symmetric triaxial configuration that is not strictly Galperin, with a tilt angle α of approximately 30° [19]. While the treatment of coordinate conversion is well established for the Galperin configuration [8,9,10,13] there has been less attention on the possible benefits of employing a non-Galperin tilt angle. In this work, we derive the analytical transformation matrix from test mass displacement in the UVW coordinate to ground displacement in the XYZ coordinates and evaluate how self-noise, which equally affects each of the U, V, and W sensors, translates into X, Y, and Z noise.

One limitation of the Galperin transformation matrix is that it was derived with the assumption of a point mass on a massless boom, while in practice, masses are distributed along the boom. Another limitation is its applicability at lower frequencies. At lower frequencies, the displacements perpendicular to the booms contains the resonance structure of the rotational mass-spring oscillator. A strict application of the Galperin transformation matrix would lead to an absurd result that the displacements on the ground also have this resonance structure. Both of these limitations do not affect the operations of seismometers when they use the torque feedback technique, which works at all frequencies and does not depend on how masses are distributed along the boom. Nonetheless, given the ubiquitous applications of the Galperin triaxial seismometers, it is useful to have a deeper understanding of how such seismometers would behave in the absence of torque feedback. This is particularly true in the commissioning phase of these seismometers, where torque feedback may be disabled to measure parameters needed to establish the noise model. Therefore, we have extended the transformation matrix to cover the case of distributed masses on the boom, and the case of lower frequency operations.

Since torque feedback is ubiquitously used, the most useful transformation matrix is one that transforms the feedback torque to ground acceleration. We derive such a transformation matrix using an extension of the equation of motion by Huang and Saulson [20] to include external torques resulting from ground acceleration.

To keep the discussion easy to understand, we assumed that the component seismometers are oriented symmetrically with 120° separations and that they are identical. After the main idea is presented in the main text, the more laborious cases of non-symmetrical orientation and non-identical component seismometers are presented in Appendix B and Appendix C.

Although we have discussed the Brownian noise and noise due to temperature sensitivity, it is not the main focus of this paper to treat all noise issues of seismometers. We used Brownian noise and temperature sensitivity noise to discuss how un-correlated noise and correlated noise propagate through the Galperin transformation. The expression for both of these noises, in the vertical direction, had already been published by Erwin et al. [21]. In the current paper, we illustrate how the Galperin transformation can be used to get the noise in the horizontal directions. For more in-depth discussions of other sources of noise in a seismometer, we refer the readers to a publication by Mimoun et al. [22].

## 2. Extension of the Transformation Matrices to Treat Arbitrary Values of *α*

Figure 1 depicts the configuration of the symmetric triaxial seismometer. The Galperin transformation transforms the test mass displacements u, v, and w (as viewed from the ground) in the U, V, and W direction into ground displacement X, Y, and Z in the X, Y, and Z direction. As already mentioned, there is an implicit assumption that the ground is moving at frequencies much higher than the resonance frequencies of the rotational mass-spring oscillator. At such higher frequencies, the test mass does not move when viewed from an inertial reference frame. Only the ground moves.

In this section, we derive the individual contributions of X, Y, and Z ground displacements on the test mass displacements u, v, and w as viewed from the ground. The individual contributions can then be summed, from which we can ultimately obtain a transformation matrix from u, v, and w to X, Y, and Z.

### 2.1. Boom Displacement Due to Vertical Ground Motion

**Figure 2:**
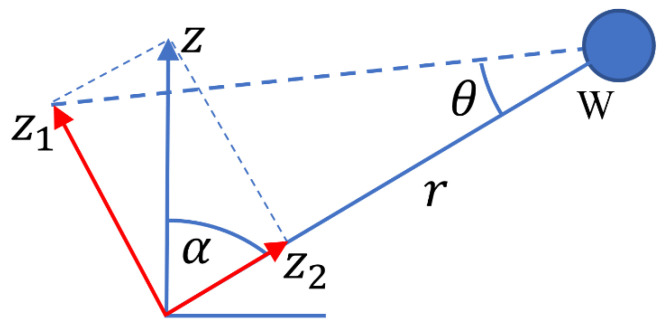
The W component seismometer is in its plane of motion, with the ground, drawn as a horizontal line, moving upward by a displacement of *z*.

Consider that the ground moves upward by a displacement Z, as depicted in Figure 2 for the W component seismometer. We can decompose the upward Z ground displacement into a component $Z_{2}$ parallel to the boom, and a component $Z_{1}$ perpendicular to it, as shown in red in Figure 2. The component $Z_{2}$ does not cause the angle α to change, while the component $Z_{1}$ causes α to increase by an angle given by $\theta =\frac{Z_{1}}{r}=\frac{Z\sin\alpha }{r}$. Since w=rθ, we have the result

(1) $$w_{z}=Z\sin\alpha ,$$

where $w_{z}$ is the displacement w caused by ground motion in the vertical direction. Similarly, for the U and V sensors, we find that the contributions due to ground motion in the Z direction are

(2) $$u_{z}=Z\sin\alpha$$

(3) $$v_{z}=Z\sin\alpha .$$

### 2.2. Boom Displacement Due to Horizontal Ground Motion

**Figure 3:**
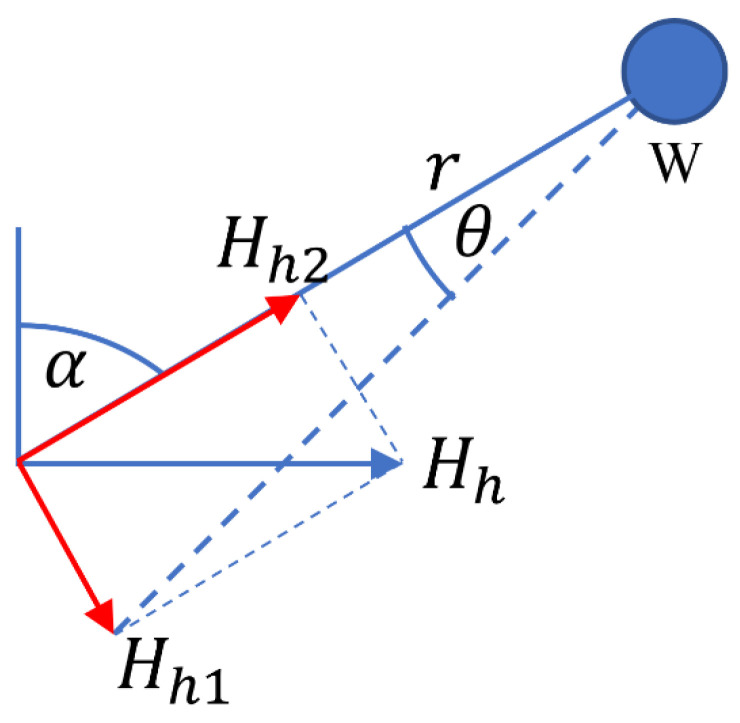
The W component seismometer in its plane of motion, with the ground moving sideward by a displacement of *H_h_* inside this plane.

Now consider that the ground moves by a horizontal displacement $H_{h}$ in the plane of rotation of the W component boom as depicted in Figure 3. The displacement $H_{h}$ can be decomposed into a component $H_{h2}$ along the direction of the boom, and a component $H_{h1}$ perpendicular to it, as shown in red in Figure 3. The component $H_{h2}$ does not cause the angle α to change, while the component $H_{h1}$ causes α to decrease. Therefore, $\theta =-\frac{H_{h1}}{r}=-\frac{h\cos\alpha }{r}$. Since w=rθ, we find that

(4) $$w_{h}=-H_{h}\cos\alpha .$$

This horizontal motion has both an x and y-axis component. To see how this translates onto the UVW sensors, we consider the case where the ground moves by a displacement in the X and Y directions separately.

*Horizontal Motion in the X direction.* If the horizontal motion is in the X direction, the ground displacement X can be decomposed into a component $X_{2}$ along the plane of motion of the W component seismometer, and a component $X_{1}$ perpendicular to this plane, as shown by the red arrows in Figure 1b. The component $X_{1}$ cannot cause the angle α to change, while the component $X_{2}$ can be identified as the displacement $H_{h}$ in Equation (4). Therefore, Equation (4) becomes

(5) $$w_{x}=-X_{2}\cos\alpha =-X\cos\alpha \sin\beta =-\frac{X\cos\alpha }{2},$$

where β=30°. Following a similar process for the V component seismometer, we have

(6) $$v_{x}=-\frac{X\cos\alpha }{2}.$$

For the U component seismometer, one can change w in Equation (4) to u and identify h as −X to obtain

(7) $$u_{x}=X\cos\alpha .$$

*Horizontal Motion in the Y direction.* Consider the case where the ground moves horizontally by a displacement Y along the y-axis. Since the motion is perpendicular to the plane of motion of the U component seismometer, it has no effect on the displacement u, hence

(8) $$u_{y}=0.$$

Applying the same process as discussed for motion in the X direction for the W component sensor, we obtain

(9) $$w_{y}=-Y\cos\alpha \cos\beta =-\frac{\sqrt{3}}{2}Y\cos\alpha ,$$

(10) $$v_{y}=Y\cos\alpha \cos\beta =\frac{\sqrt{3}}{2}Y\cos\alpha .$$

### 2.3. Symmetric Triaxial Transformation Matrices

Since the displacement of a test mass is the sum of its displacements resulting from ground motion in the X, Y, and Z directions, we have $u=u_{x}+u_{y}+u_{z}$, $v=v_{x}+v_{y}+v_{z}$, and $w=w_{x}+w_{y}+w_{z}$. Arranging these three formulas in matrix notation, we arrive at the transformation matrix from XYZ to UVW signals

(11) $$\left[\begin{array}{c} u\\ v\\ w \end{array}\right]=\left[\begin{array}{ccc} \cos\alpha & 0 & \sin\alpha \\ -\frac{\cos\alpha }{2} & \frac{\sqrt{3}}{2}\cos\alpha & \sin\alpha \\ -\frac{\cos\alpha }{2} & -\frac{\sqrt{3}}{2}\cos\alpha & \sin\alpha \end{array}\right]\left[\begin{array}{c} X\\ Y\\ Z \end{array}\right].$$

In Appendix B, we present a more general form of this matrix with arbitrary angles.

Inverting this matrix, we obtain the conversion from UVW to XYZ

(12) $$\left[\begin{array}{c} X\\ Y\\ Z \end{array}\right]=\left[\begin{array}{ccc} \frac{2}{3\cos\alpha } & -\frac{1}{3\cos\alpha } & -\frac{1}{3\cos\alpha }\\ 0 & \frac{1}{\sqrt{3}\cos\alpha } & -\frac{1}{\sqrt{3}\cos\alpha }\\ \frac{1}{3\sin\alpha } & \frac{1}{3\sin\alpha } & \frac{1}{3\sin\alpha } \end{array}\right]\left[\begin{array}{c} u\\ v\\ w \end{array}\right].$$

The transformation matrices in Equations (11) and (12) are valid for any tilt angle α. One should note that the transformation matrix may differ slightly in the signs of its elements depending on how the UVW axis and the XYZ axis are arranged with respect to one another.

As a check on our matrices, we substitute for the Galperin configuration, $\alpha =\tan^{-1}\frac{1}{\sqrt{2}}$, into Equations (11) and (12). Making this substitution, we obtain the Galperin transformation matrices generally cited in the literature [8,9], where

(13) $$\left[\begin{array}{c} u\\ v\\ w \end{array}\right]=\left[\begin{array}{ccc} \sqrt{2/3} & 0 & 1/\sqrt{3}\\ -1/\sqrt{6} & 1/\sqrt{2} & 1/\sqrt{3}\\ -1/\sqrt{6} & -1/\sqrt{2} & 1/\sqrt{3} \end{array}\right]\left[\begin{array}{c} X\\ Y\\ Z \end{array}\right],$$

and

(14) $$\left[\begin{array}{c} X\\ Y\\ Z \end{array}\right]=\left[\begin{array}{ccc} \sqrt{2/3} & -1/\sqrt{6} & -1/\sqrt{6}\\ 0 & 1/\sqrt{2} & -1/\sqrt{2}\\ 1/\sqrt{3} & 1/\sqrt{3} & 1/\sqrt{3} \end{array}\right]\left[\begin{array}{c} u\\ v\\ w \end{array}\right].$$

We acknowledge that Equation (11) is the same as the one presented by Peng, Xue, and Yang [10]. However, in their analysis, they used numerical transformation instead of the analytical form given by Equation (12).

## 3. Noise Conversions from UVW to XYZ Coordinates

In this section, we use the preceding transformation matrix to study how to instrument noise from the seismometer’s U, V, and W sensors propagate into ground displacement noise in the X, Y, and Z directions. We pay particular attention to how uncorrelated noises propagate as opposed to those of correlated noises. From Equation (12), we find that

(15) $$X=\frac{1}{3\cos\alpha }\left(2u-v-w\right),$$

(16) $$Y=\frac{1}{\sqrt{3}\cos\alpha }\left(v-w\right),$$

(17) $$Z=\frac{1}{3\sin\alpha }\left(u+v+w\right).$$

### 3.1. Uncorrelated Noise Sources

We use the notation $\sigma_{u}$ to represent the root mean square value of u. We consider instrument noise that affects each sensor equally, i.e., $\sigma_{u}=\sigma_{v}=\sigma_{w}$. We first assume that the noise in u, v, and w are not correlated. Uncorrelated noises include Brownian noise and electronic noises from the displacement capacitance sensors. Applying the rule for the propagation of uncorrelated noise to Equations (15)–(17), we obtained

(18) $$\sigma_{X}=\frac{\sqrt{4\sigma_{u}^{2}+\sigma_{v}^{2}+\sigma_{w}^{2}}}{3\cos\alpha }=\sqrt{\frac{2}{3}}\frac{\sigma_{u}}{\cos\alpha }\;,$$

(19) $$\sigma_{Y}=\frac{\sqrt{\sigma_{v}^{2}+\sigma_{w}^{2}}}{\sqrt{3}\cos\alpha }=\sqrt{\frac{2}{3}}\frac{\sigma_{u}}{\cos\alpha }\;,$$

(20) $$\sigma_{Z}=\frac{\sqrt{\sigma_{u}^{2}+\sigma_{v}^{2}+\sigma_{w}^{2}}}{3\sin\alpha }=\frac{\sigma_{u}}{\sqrt{3}\sin\alpha }.$$

It is interesting to consider the cases where Equations (18)–(20) diverge and converge. For the case where the boom is aligned with the vertical axis (α=0), all three component seismometers have no sensitivity to vertical ground motion; Equation (20) diverges while Equations (18) and (19) converge to $\sigma_{u}\sqrt{2/3}$. On the other hand, when all three booms are horizontal (α=π/2), all three component seismometers have no sensitivity to horizontal ground motion; Equations (18) and (19) diverge whereas Equation (20) converges to $\sigma_{u}/\sqrt{3}$.

### 3.2. Correlated Noise Sources

There are noise sources that are correlated. Noise induces by random temperature variation typically affects all three component seismometers together. For completely correlated noises, Equations (15)–(17) predict that

(21) $$\sigma_{X}=\sigma_{Y}=0,$$

(22) $$\sigma_{z}=\frac{\sigma_{u}}{\sin\alpha }\;.$$

It is interesting to note that the Galperin transformation has the effect of suppressing correlated noise in the X and Y directions.

### 3.3. Noise Factor

In this subsection, we define a metric for evaluating how uncorrelated horizontal and vertical ground noise vary as a function of tilt angle. We define a horizontal noise factor as $N_{H}=N_{x}=N_{y}=\sigma_{x}/\sigma_{u}$, and a vertical noise factor as $N_{V}=\sigma_{z}/\sigma_{u}$. From Equations (18) and (20), we have

(23) $$N_{H}=\sqrt{\frac{2}{3}}\frac{1}{\cos\alpha }\;,$$

(24) $$N_{V}=\frac{1}{\sqrt{3}\sin\alpha }\;.$$

**Figure 4:**
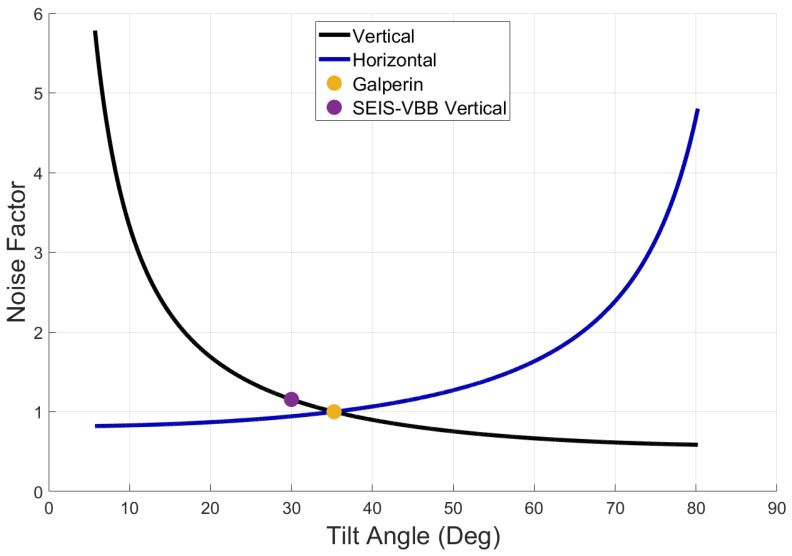
The dimensionless noise factor for a triaxial seismometer for reporting a single horizontal or vertical axis.

In Figure 4, both noise factors are plotted as a function of tilt angle. For the Galperin configuration ($\alpha =\tan^{-1}1/\sqrt{2}$), both noise factors are 1. For the Very Broadband Seismometer of the Insight mission to Mars, α≈29.5° and the noise factor is 1.15 in the vertical, which is exactly what Lognonné et al. (2019) reported.

## 4. The Concept of Null Point

In the preceding derivation, the seismometer is assumed to be a point mass on a massless boom, while in any real seismometer, the mass is distributed along the boom, in which case there is no clear location along the boom where the displacements u, v, and w should be evaluated. If it is not evaluated at the correct location, the transformation matrix will give the wrong answer for ground displacements. Conventional wisdom may lead us to use the location of the center of mass for the evaluation. Nevertheless, we will give an example to show that this is wrong.

In the preceding derivation, we assume that the ground moves at a frequency much higher than that of the resonance frequency of the mass-spring oscillator. In such a case, relative to an inertial frame, the test mass does not move, only the ground moves. For distributed mass on a boom, we expect that there is also a point on the boom which does not move, while the rest of the boom, as well as the ground move. We call this point the Null Point. The Null Point is the location where the displacement of a component seismometer should be evaluated because it will give a well-defined angular deflection of the boom when the ground moves up and down. If the ground moves up and down by a displacement Z, then the angle of deflection increases and decreases by

(25) $$\theta =Z\sin\alpha /D_{n},$$

where $D_{n}$ is the distance between the Null Point and the pivot. To an observer on the ground (which is an accelerating frame), the Null Point would move up and down by the same displacement Z, and the angle α would increase and decrease by the same θ. This is the same θ(t) that should be used in the equation of motion of the system. The link between the equation of motion and the transformation matrix is therefore

(26) $$u=D_{n}\theta .$$

**Figure 5:**
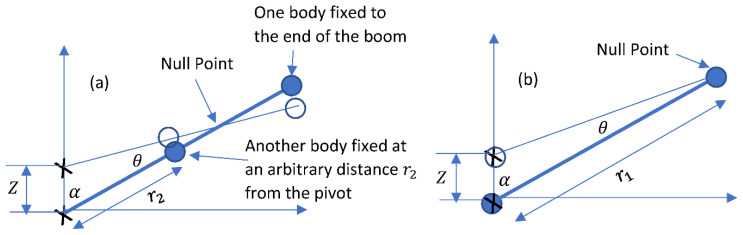
(**a**) Two identical bodies on a massless boom. When the ground moves up and down at high frequencies, there is a location on the boom that does not move. We call this location the Null Point. (**b**) When the movable mass is placed at or near the location of the pivot, it moves up and down with the ground and does not contribute to changing the angle *θ*. In this case, the Null Point moves back to the end of the boom.

Now, consider the example shown in Figure 5, where there are two bodies of the same mass $m_{1}$ on a massless boom. This is the simplest example of distributed masses on a boom. One body is always at the end of the boom of length $r_{1}$, while the other can be placed anywhere along the boom. We assume a variable distance $r_{2}$ between the center of mass of the movable body and the pivot. When the movable body is also at the end of the boom, the Null Point will also be at the end of the boom, such as in the case discussed before with point mass. However, when the movable body is moved all the way to the pivot, it will move up and down with the pivot, which is attached to the ground. It will not contribute to the deflection of the boom. In this case, the Null point will also be at the end of the boom, and not at the location of the center of mass of the combined two bodies.

## 5. Extension of the Equation of Motion to Include Ground Acceleration

To derive a formula for $D_{n}$, we follow the derivation of Erwin et al. [21] for the torque on the boom due to vertical ground acceleration. The static torque on the boom is

(27) $$\Gamma =mgD_{g}\sin\alpha ,$$

where g is the local gravitational acceleration, m and $D_{g}$ are the suspended mass and its center of mass respectively. An observer on an accelerating platform (the ground) with an upward acceleration of $\ddot{Z}$ would feel an additional downward acceleration of the same value, as though the local gravity had increased. Therefore, the torque due to vertical ground acceleration is

(28) $${\delta \Gamma }_{z}=mD_{g}\sin\alpha \delta g=mD_{g}\sin\alpha \ddot{Z}.$$

Similarly, if the ground accelerated horizontally to the right along a direction h in the plane of motion of the boom, and if the horizontal acceleration is $\ddot{H_{h}}$, the masses on the boom would feel an equivalent gravitational pull of $\ddot{-H_{h}}$ on it. The additional torque on the boom would be

(29) $${\delta \Gamma }_{h}=-mD_{g}\cos\alpha {\ddot{H}}_{h}.$$

The derivations of Equations (28) and (29) are based on Einstein’s equivalence principle, which states that an observer on an enclosed accelerating platform cannot tell if he is on the acceleration platform or is being pulled by gravity on a stationary platform. This implies that the acceleration of the platform can be treated as an equivalent additional gravitational acceleration by an observer on the platform. Adding these torques as additional torques to the equation of motion of a rotational mass-spring oscillator as given by Huang and Saulson (1994), we obtain

(30) $$J\ddot{\theta }+\left[K_{s}\left(1+i\phi \right)-mgD_{g}\cos\alpha \right]\theta +B\dot{\theta }=mD_{g}\sin\alpha \ddot{Z}-mD_{g}\cos\alpha {\ddot{H}}_{h},$$

where J is the moment of inertia, $K_{s}$ is the rotational spring rate, ϕ is the loss angle of spring material, and B is the viscous damping coefficient. The imaginary unit i is used to represent losses in the equation.

## 6. Using the Equation of Motion to Derive *D_n_*

When the angular frequency ω is much higher than the angular resonance frequency $\omega_{o}$, the $\ddot{\theta }$ term dominates over the θ and $\dot{\theta }$ term because $\left|\ddot{\theta }\right|=\omega^{2}\left|\theta \right|$, while the $\dot{\theta }$ is only proportional to ω. If we also neglect horizontal acceleration, the equation of motion simplifies to

(31) $$J\ddot{\theta }=mD_{g}\sin\alpha \ddot{Z},$$

or by integrating twice,

(32) $$\theta =Z\sin\alpha /D_{1},$$

where $D_{1}=\frac{J}{mD_{g}}$.

Comparing Equation (32) to Equation (25), we found that $D_{1}=D_{n}$. Therefore,

(33) $$D_{n}=\frac{J}{mD_{g}}.$$

**Figure 6:**
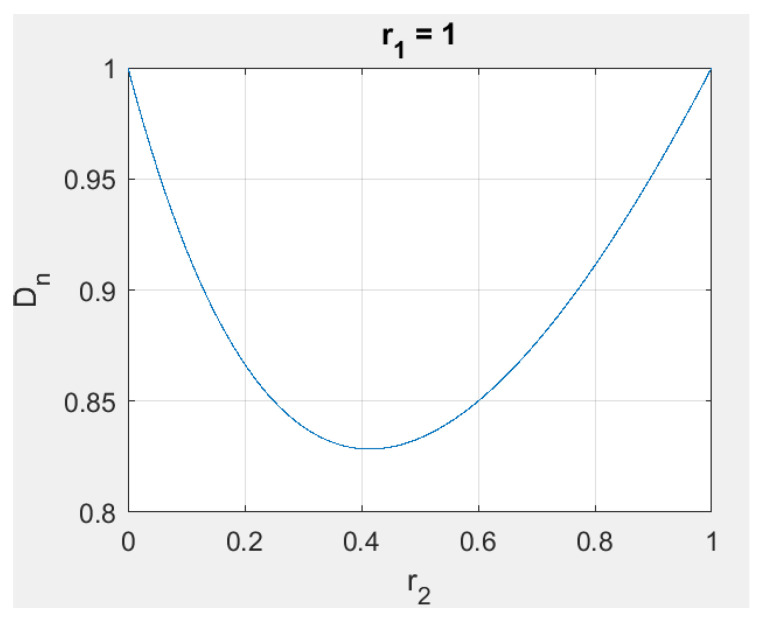
The Null Point location as a function of the distance of the movable body from the pivot.

For example, as shown in Figure 5, $D_{n}=\frac{r_{1}^{2}+r_{2}^{2}}{r_{1}+r_{2}}$. A plot of $D_{n}$ vs. $r_{2}$ is shown in Figure 6 for $r_{1}=1$. The behavior of $D_{n}$ is consistent with our intuitive discussion that the Null Point should be at the end of the boom when the movable body is moved all the way to the location of the pivot.

## 7. Extension of the Transformation Matrices for Operations at All Frequencies

The transformation matrices given in Equations (11) and (12) are valid only when $\omega \gg \omega_{o}$. With the help of the concept of a Null Point, we can extend the transformation to cover operations at all frequencies. In the frequency domain, the equation of motion given by Equation (30) becomes

(34) $$H_{r}\left(\omega \right)\theta_{\omega }=-\omega^{2}\left(mD_{g}/J\right)\left(\sin\alpha Z_{\omega }-\cos\alpha H_{h\_\omega }\right),$$

where the transfer function $H_{r}\left(\omega \right)=-\omega^{2}+\left(K_{s}-mgD_{g}\cos\alpha \right)/J+i\left(\omega_{s}^{2}\phi +\omega B/J\right),$ or

(35) $$H_{r}\left(\omega \right)=\left(\omega_{o}^{2}-\omega^{2}\right)+i\left(\omega_{s}^{2}\phi +\omega B/J\right),$$

where the reduced angular spring constant is $K_{o}=K_{s}-mgD_{g}\cos\alpha$, and the angular resonance frequencies are $\omega_{o}=\sqrt{K_{o}/J}$, $\omega_{s}=\sqrt{K_{s}/J}$ and we have used the notation that a subscript of ω on a quantity represents the normalized Fourier transform of that quantity. In this notation, the power spectral density of *θ* is ${\left|\theta_{\omega }\right|}^{2}$, where $\theta_{\omega }$ is normalized in a way that the Parseval theorem is obeyed i.e., $\theta_{rms}^{2}=\int_{0}^{\infty }{\left|\theta_{\omega }\right|}^{2}d\omega$. We apply Equation (34) to the W component seismometer by using $w=D_{n}\theta$ from Equation (26). We then multiply Equation (34) by $D_{n}/H_{r}\left(\omega \right)$, and apply $D_{n}=J/\left(mD_{g}\right)$ from Equation (32) we obtain

(36) $$w_{\omega }=\frac{-\omega^{2}}{H_{r}\left(\omega \right)}\left(\sin\alpha Z_{\omega }-\cos\alpha H_{h\_\omega }\right),$$

For a triaxial seismometer, one can decompose the displacement in each of the U, V, and W seismometers as a result of ground acceleration in the X, Y, and Z directions. For example, if $w_{\omega z}$ is the displacement in the W seismometer due to ground acceleration in the Z direction, then from Equation (36)

(37) $$w_{z\omega }=\frac{-\omega^{2}}{H_{r}\left(\omega \right)}\sin\alpha Z_{\omega }.$$

Similarly, from Equation (36) the displacement in the W seismometer due to ground acceleration in the horizontal direction in the plane of rotation is

(38) $$w_{h\omega }=\frac{\omega^{2}}{H_{r}\left(\omega \right)}\cos\alpha H_{h\_\omega }.$$

Comparing Equation (37) to Equation (1), one notices that if one replaces $w_{z}$ and Z in Equation (1) with $w_{z\omega }$ and $\frac{-\omega^{2}}{H_{r}\left(\omega \right)}Z_{\omega }$ respectively, Equation (1) will turn into Equation (37). Similarly, if one replaces $w_{h}$ and $H_{h}$ in Equation (4) with $w_{z\omega }$ and $\frac{-\omega^{2}}{H_{r}\left(\omega \right)}H_{h\_\omega }$ respectively, Equation (4) will turn into Equation (38). Due to the one-to-one correspondence, one can write the frequency domain transformation for any frequency as

(39) $$\left[\begin{array}{c} u_{\omega }\\ v_{\omega }\\ w_{\omega } \end{array}\right]=-\frac{\omega^{2}}{H_{r}\left(\omega \right)}\left[\begin{array}{ccc} \cos\alpha & 0 & \sin\alpha \\ -\frac{\cos\alpha }{2} & \frac{\sqrt{3}}{2}\cos\alpha & \sin\alpha \\ -\frac{\cos\alpha }{2} & -\frac{\sqrt{3}}{2}\cos\alpha & \sin\alpha \end{array}\right]\left[\begin{array}{c} X_{\omega }\\ Y_{\omega }\\ Z_{\omega } \end{array}\right],$$

where the measured displacement in the U seismometer is $u_{\omega }=u_{x\omega }+u_{y\omega }+u_{z\omega }$. Similarly, $v_{\omega }=v_{x\omega }+v_{y\omega }+v_{z\omega }$, and $w_{\omega }=w_{x\omega }+w_{y\omega }+w_{z\omega }$. Inverting this, we obtain

(40) $$\left[\begin{array}{c} X_{\omega }\\ Y_{\omega }\\ Z_{\omega } \end{array}\right]=-\frac{H_{r}\left(\omega \right)}{\omega^{2}}\left[\begin{array}{ccc} \frac{2}{3\cos\alpha } & -\frac{1}{3\cos\alpha } & -\frac{1}{3\cos\alpha }\\ 0 & \frac{1}{\sqrt{3}\cos\alpha } & -\frac{1}{\sqrt{3}\cos\alpha }\\ \frac{1}{3\sin\alpha } & \frac{1}{3\sin\alpha } & \frac{1}{3\sin\alpha } \end{array}\right]\left[\begin{array}{c} u_{\omega }\\ v_{\omega }\\ w_{\omega } \end{array}\right].$$

At high frequencies, $H_{r}\left(\omega \right)\to \omega^{2}$, Equations (39) and (40) becomes Equations (11) and (12).

In Equations (39) and (40), the displacement u, v and w are evaluated at the Null Point. However, one can easily evaluate the displacement at the sensor position by replacing u, v and w in Equations (39) and (40) with $u{}^{\prime }D_{n}/D_{c}$, $v{}^{\prime }D_{n}/D_{c}$ and $w{}^{\prime }D_{n}/D_{c}$ respectively where $u{}^{\prime }$, $v{}^{\prime }$ and $w{}^{\prime }$ are the displacement measured at the motion sensor and $D_{c}$ is the distance from the motion sensor to the pivot.

The time domain transformation from u(t), v(t), and w(t) to $\ddot{X}\left(t\right)$, $\ddot{Y}\left(t\right)$, and $\ddot{Z}\left(t\right)$ is more complicated. It is discussed in Appendix E.

## 8. Extension of the Transformation Matrices for Torque Feedback Operations

Generally, the triaxial seismometers are operated in a torque feedback mode, which gives valid results for all frequencies.

In the presence of externally applied torque, the equation of motion (Equation (30)) becomes

(41) $$\Gamma_{ext}=J\ddot{\theta }+\left[K_{s}\left(1+i\phi_{i}\right)-mgD_{g}\cos\alpha \right]\theta +B\dot{\theta },$$

where the externally applied torque $\Gamma_{ext}$ includes the Brownian noise torque $\Gamma_{B}$, the feedback torque $\Gamma_{FB}$, and the torque due to ground motion $\Gamma_{G}$, i.e., $\Gamma_{ext}=\Gamma_{B}+\Gamma_{FB}+\Gamma_{G}$, and $\Gamma_{G}=mD_{g}\sin\alpha \ddot{Z}-mD_{g}\cos\alpha {\ddot{H}}_{h}.$ Consider the case where torque feedback is used to keep θ close to zero, and Brownian torque is negligible. In this case, for a single-component seismometer, the equation of motion (Equation (41)) becomes

(42) $$\Gamma_{FB}=-\Gamma_{G}=-mD_{g}\sin\alpha \ddot{Z}+mD_{g}\cos\alpha {\ddot{H}}_{h}$$

For a triaxial seismometer, one can decompose $\Gamma_{FB}$ into component torques in each of the U, V, and W seismometers as a result of ground acceleration in X, Y, and Z directions. For example, if $\Gamma_{wz}$ is the feedback torque in the W seismometer due to ground acceleration in the Z direction, then from Equation (42)

(43) $$\Gamma_{wz}=-mD_{g}\sin\alpha \ddot{Z}.$$

Similarly, $\Gamma_{uz}=\Gamma_{vz}=-mD_{g}\sin\alpha \ddot{Z}.$ Similarly, the feedback torque in the W seismometer due to ground acceleration in the horizontal direction in the plane of rotation is

(44) $$\Gamma_{wh}=mD_{g}\cos\alpha {\ddot{H}}_{h}.$$

Using the same argument of one-to-one correspondence of equations to those in Equation (1) through Equation (10), we found that

(45) $$\left[\begin{array}{c} \Gamma_{u}\\ \Gamma_{v}\\ \Gamma_{w} \end{array}\right]=-mD_{g}\left[\begin{array}{ccc} \cos\alpha & 0 & \sin\alpha \\ -\frac{\cos\alpha }{2} & \frac{\sqrt{3}}{2}\cos\alpha & \sin\alpha \\ -\frac{\cos\alpha }{2} & -\frac{\sqrt{3}}{2}\cos\alpha & \sin\alpha \end{array}\right]\left[\begin{array}{c} \ddot{X}\\ \ddot{Y}\\ \ddot{Z} \end{array}\right],$$

where the measured feedback torque in the U seismometer is $\Gamma_{u}=\Gamma_{ux}+\Gamma_{uy}+\Gamma_{uz}$. Similarly, $\Gamma_{v}=\Gamma_{vx}+\Gamma_{vy}+\Gamma_{vz}$, and $\Gamma_{w}=\Gamma_{wx}+\Gamma_{wy}+\Gamma_{wz}$.

Inverting the matrix, we obtain the conversion matrix from measuring feedback torques to ground accelerations as.

(46) $$\left[\begin{array}{c} \ddot{X}\\ \ddot{Y}\\ \ddot{Z} \end{array}\right]=-\frac{1}{mD_{g}}\left[\begin{array}{ccc} \frac{2}{3\cos\alpha } & -\frac{1}{3\cos\alpha } & -\frac{1}{3\cos\alpha }\\ 0 & \frac{1}{\sqrt{3}\cos\alpha } & -\frac{1}{\sqrt{3}\cos\alpha }\\ \frac{1}{3\sin\alpha } & \frac{1}{3\sin\alpha } & \frac{1}{3\sin\alpha } \end{array}\right]\left[\begin{array}{c} \Gamma_{u}\\ \Gamma_{v}\\ \Gamma_{w} \end{array}\right].$$

In Appendix D, we generalize this matrix to cover the case of non-identical component seismometers that are not separated 120° appart.

Notice that the concept of Null Point is not needed in this derivation. Since the torque feedback technique is most frequently used, the necessity of the concept of the Null Point was never realized.

## 9. Brownian Noise

As an example of how to use our transformation matrices, we demonstrate how the Brownian noise in the vertical direction presented by Erwin et al. [21] can be extended to give Brownian noise in the x and y directions. The seismometer output noise in the vertical direction due to Brownian motion is given by Erwin et al. [21] as

(47) $$\left|A_{B\_z\_\omega }\right|=\frac{\left|\Gamma_{B\_\omega }\right|}{\sqrt{3}mD_{g}\sin\alpha }\;,$$

where the Brownian torque noise spectral density is

(48) $$\left|\Gamma_{B\_\omega }\right|=\sqrt{4k_{B}T\left(\frac{K_{s}\phi }{\omega }+B\right)}\;,$$

and B is the viscous damping coefficient. This Brownian torque noise is a translation from the Brownian force noise in a linear mass-spring oscillator presented by Erwin et al. [23].

In the following, we shall define A as the acceleration output of the triaxial seismometer, $A_{B}$ as the Brownian noise component of this output.

If the torque due to ground acceleration is substantially smaller than Brownian torque noise, the measured feedback torque would be predominantly the Brownian torque. Since the seismometer would make use of Equation (46) to calculate ground acceleration in the X, Y, and Z directions. It would misinterpret this Brownian torque as ground acceleration. The seismometer’s acceleration output in the time domain would therefore be

(49) $$\left[\begin{array}{c} A_{B\_x}\\ A_{B\_y}\\ A_{B\_z} \end{array}\right]=\frac{1}{mD_{g}}\left[\begin{array}{ccc} \frac{2}{3\cos\alpha } & -\frac{1}{3\cos\alpha } & -\frac{1}{3\cos\alpha }\\ 0 & \frac{1}{\sqrt{3}\cos\alpha } & -\frac{1}{\sqrt{3}\cos\alpha }\\ \frac{1}{3\sin\alpha } & \frac{1}{3\sin\alpha } & \frac{1}{3\sin\alpha } \end{array}\right]\left[\begin{array}{c} \Gamma_{Bu}\\ \Gamma_{Bv}\\ \Gamma_{Bw} \end{array}\right],$$

where $\Gamma_{Bu}$ is the Brownian torque in the U seismometer. In the frequency domain, $\Gamma_{Bu_{\omega }}$, $\Gamma_{Bv_{\omega }}$ and $\Gamma_{Bw_{\omega }}$ are uncorrelated with a magnitude given by Equation (48). We use the rule for error propagation for uncorrelated signals and obtained the Brownian noise spectrum in the X and Y directions as

(50) $$\left|A_{B\_x\_\omega }\right|=\left|A_{B\_y\_\omega }\right|=\frac{\sqrt{2/3}\left|\Gamma_{B\_\omega }\right|}{mD_{g}\cos\alpha },$$

and the same $\left|A_{B\_z\_\omega }\right|$ as given by Erwin et al. [21] and shown in Equation (47).

One should also note that for a single-component seismometer, the output Brownian noise in the vertical and horizontal directions are $\frac{\left|\Gamma_{B\_\omega }\right|}{mD_{g}\sin\alpha }$ and $\frac{\left|\Gamma_{B\_\omega }\right|}{mD_{g}\cos\alpha }$ respectively. One should also note that these two noises are completely correlated because the rotational mass-spring oscillator has only one degree of thermodynamic freedom. These two noises must originate from the same degree of freedom and therefore must be correlated.

In Appendix F, we present another way to derive the Brownian noise in the vertical direction and show that it is consistent with Equation (47) only if the Null Point distance $D_{n}$ is used. If other distances such as the location of the displacement sensor were used, it would lead to an inconsistent result. This illustrates the importance of the concept of the Null Point in making the physics of the rotational mass-spring oscillator consistent.

## 10. Temperature Sensitivity

The usefulness of our transformation matrix can also be illustrated by extending the temperature sensitivity in the vertical direction as derived by Erwin et al. [21] to cover the temperature sensitivities in the X and Y directions. Erwin et al. [21] showed that the temperature sensitivity in the vertical direction is

(51) $$\frac{dA_{z}}{dT}=g\left[\alpha_{CTE}-\beta_{o}+\cot\alpha {\left(\frac{d\alpha_{o}}{dT}\right)}_{\omega_{o}}+\frac{K_{o}}{mD_{g}g\sin\alpha }{\left(\frac{d\alpha_{o}}{dT}\right)}_{\omega_{o}}\right]\;,$$

where the relative coefficient of thermal expansion of the boom is $\alpha_{CTE}=\frac{1}{D_{g}}\frac{dD_{g}}{dT}$, and $\beta_{o}=\frac{1}{K_{s}}\frac{dK_{s}}{dT}$ is the relative thermoelastic coefficient of the spring, $\alpha_{o}$ is the angle between the boom and the vertical in the absence of gravity, and $K_{o}$ is the angular spring rate when the frequency of the oscillator is reduced by mechanical or electrostatic means. Following the derivation of Erwin et al. [21], we obtained the temperature sensitivity in the horizontal direction in the plane of rotation as

(52) $$\frac{dA_{h}}{dT}=g\left[\tan\alpha \left(\alpha_{CTE}-\beta_{o}\right)+{\left(\frac{d\alpha_{o}}{dT}\right)}_{\omega_{o}}+\frac{K_{o}}{mD_{g}g\cos\alpha }{\left(\frac{d\alpha_{o}}{dT}\right)}_{\omega_{o}}\right]\;.$$

Since the U, V, and W component seismometers are constructed the same way, they are likely to have close to the same temperature sensitivity. Assuming that their temperature sensitivities are the same and that the temperature differences between them are small compared to the temperature excursion, then the error signal due to temperature changes would be largely correlated. Assuming perfect correlation, Equation (46) or Equation (21) gives

(53) $$\frac{dA_{x}}{dT}=\frac{dA_{y}}{dT}=0,$$

and $\frac{dA_{z}}{dT}$ is the same as that of a single-component seismometer given by Equation (51). It is rather surprising to find that the ideal symmetric triaxial seismometer’s output in the X and Y direction is not sensitive to temperature change under ideal conditions. In practice, the assumptions stated are not met to a certain extent. The thermal sensitivities of the U, V, and W seismometer components are not identical. The temperature perturbations experienced by U, V, and W are not identical. For example, solar radiation hits the seismometer from one side which leads to lateral temperature gradients. Inhomogeneity of the thermal insulation and inhomogeneity of the thermal properties of the sensor and the soil on which it rests, all lead to non-identical temperature noise at the three component seismometers. However, based on the results from the ideal case, one should expect a significant reduction in temperature sensitivity compared to that of a single-component seismometer in the horizontal direction (see Equation (52)). The zero sensitivity in the ideal case should also motivate developers of the next-generation seismometers to minimize non-ideal effects that cause the temperature sensitivity to be non-zero in the X and Y directions.

## 11. Discussion

When considering the seismometer self-noise, the noise formulas predict infinite noise for the case where a seismometer is tilted at such an angle that it cannot measure motion in the desired direction. The concept of infinite noise requires some explanation. We start by considering α→0 then, $\sigma_{z}\to \infty$. To show that this is correct, one can hypothetically consider the opposite case, where the noise is finite when α approaches zero. What it would mean is that such a seismometer, with the boom pointing upward, would be able to measure z component ground displacement to within some degree of uncertainty. This would contradict our intuition that such a seismometer would not be able to measure ground motion in the vertical direction at all.

In the literature, the transformation matrix is not usually presented analytically, and instead is given numerically [8,9]. In the work of Graizer (2009) the matrix is given analytically, but if we used the same approach to consider noise, the matrix would lead us to find that when α→0 then, $\sigma_{z}\to 0$ (see Appendix A), which goes against our intuition. The matrix in Graizer was likely derived by taking the transpose of Equation (11), which is valid for the Galperin configuration in which UVW leads to an orthogonal matrix, but no longer holds for non-Galperin angles.

The triaxial seismometer considered here was evaluated for the idealized case in which each sensor had an identical tilt angle and equal spacing in the horizontal, as is standard in the literature (Graizer, 2009; Wielandt, 2002; Townsend, 2014). In practice, due to manufacturing tolerance and levelling capabilities, each sensor has a unique tilt angle (see Lognonné et al., 2019). This requires the data scientist to account for the individuality of each sensor. In the appendices, we present a general transformation matrix, which accounts for misalignments of the various angles in the system, as well as component seismometers that are not identical.

## 12. Summary

We have filled in several gaps in our understanding of the Galperin triaxial seismometers. (1) We extended the Galperin transformation matrix to cover arbitrary values of the angle α. (2) We extended the Galperin transformation matrix to cover the more realistic case where masses can be distributed along the boom. We introduced the concept of Null Point to help with the understanding of how distributed masses should be treated. (3) We presented an equation of motion for a rotational mass-spring oscillator under the influence of ground acceleration in both the vertical and horizontal directions. (4) With this equation of motion, we derived the formula for $D_{n}$—the distance between the pivot and the Null Point. (5) With the help of this formula and our equation of motion, we extended the Galperin transformation matrix to cover the case of lower-frequency signals. (6) Our equation of motion also allows us to extend the transformation matrix to cover the case of torque feedback, which is the mode of operation used by nearly all seismometers. (7) We applied our transformation matrix to understand how uncorrelated noise propagates from the sensors to the acceleration output of the seismometer. With this, we extended the output noise of a triaxial seismometer in the vertical direction due to Brownian noise by Erwin et al. [21], to cover output noises in the X and Y directions. (8) We also applied our transformation matrix to understand how correlated noises propagate. Since the three component seismometers are sitting on the same thermally isolated platform, we expect that the temperature noise they experienced is largely correlated. Assuming the ideal case of complete correlation and identical component seismometers, we found a surprising result that these noises cancel out in the X and Y directions. In a more realistic case of incomplete correlation, we still expect the temperature sensitivity in the X and Y directions of a triaxial seismometer to be much smaller than the horizontal temperature sensitivity of a single component seismometer. (9) We have also derived a formula for the horizontal temperature sensitivity of a single-component seismometer. (10) In the appendices, we have further extended these matrices to cover cases of non-symmetric and non-identical component seismometers. Finally, a deeper understanding of the physics of this type of seismometer will help in the development of more sensitive ones for future planetary exploration.

## Figures and Tables

**Figure A1:**
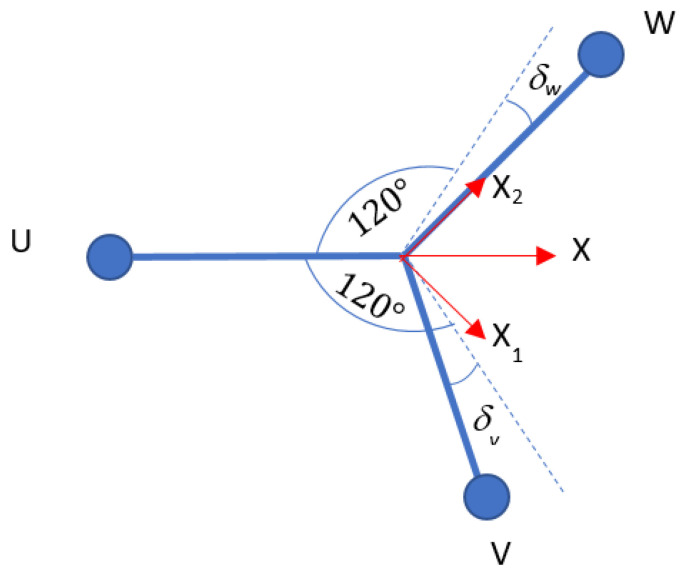
Top view of the triaxial seismometer where V and W component seismometer is not aligned with the ideal symmetric orientations shown by the dashed lines but deviate from them by angles of *δ_v_* and *δ_w_* respectively.

## Data Availability

Not applicable.

## Appendix A. Transformation Matrix Reported in the Literature

In the work of Graizer (2009), the following transformation matrix is provided from U, V, and W components to X, Y, and Z coordinates:

(A1) $$\left[\begin{array}{c} X\\ Y\\ Z \end{array}\right]=\left[\begin{array}{ccc} -\cos\alpha & \cos\alpha \sin\beta & \cos\alpha \sin\beta \\ 0 & \cos\alpha \cos\beta & -\cos\alpha \cos\beta \\ \sin\alpha & \sin\alpha & \sin\alpha \end{array}\right]\left[\begin{array}{c} u\\ v\\ w \end{array}\right],$$

where β is 30° for the symmetric seismometer. For the Galperin configuration (which was the type of arrangement Graizer considered), after accounting for discrepancies in convention for the X and Y axes, Equation (A1) yields the same numerical values as in Equation (14) and elsewhere in the literature [8,9] with the exception of some differences in the positive and negative signs in the matrix elements. These sign differences are due to two different conventions of how the *X*, *Y*, and *Z* axis are oriented relative to the *u*, *v*, and *w* axis, as explained by Townsend [9]. The transformation is not valid though for non-Galperin angles.

To illustrate this point, using Equation (A1) for the vertical direction

(A2) Z=(u+v+w)sinα

and so, the *z*-axis instrument noise for cases in which each axis experiences the same noise, $\sigma_{u}=\sigma_{v}=\sigma_{w}$, is given by

(A3) $$\sigma_{z}=\sqrt{3}\sin\alpha \sigma_{u}.$$

Now consider the goal of minimizing vertical instrument noise. Equation (A3) suggests that to minimize instrument noise in the vertical direction α should be 0°, but in this situation, the boom is pointing directly upward, and such a seismometer cannot measure Z-direction ground motion. On the other hand, equation (20) suggests that for α=0° the *z*-axis noise will be infinite, in agreement with a sensor only sensitive to horizontal ground motion.

## Appendix B. Transformation Matrix with Misalignments in the Angles

In this appendix, we derive the Galperin transformation matrix with arbitrary angles. Let $\alpha_{u}$, $\alpha_{v}$ and $\alpha_{w}$ be the angle between the boom and the vertical for the U, V, and W component seismometer respectively. Figure A1 shows the top view of the triaxial seismometer. Let the horizontal projection of the boom of the U seismometer be aligned perfectly with the −x axis. The two dashed lines are at +120° and −120° angles from the −x axis. They represent the perfect alignment locations of the W and V component seismometers. However, due to imperfections in the construction, the horizontal projections of the W and V seismometers deviate from the perfect alignment angle by angles of $\delta_{w}$ and $\delta_{v}$ respectively. Let $u_{z}$, $v_{z}$, and $w_{z}$ be the u, v and w displacements due to a vertical ground displacement of Z. Following the derivation of Equations (1), (2), and (3), we obtained

(A4) $$u_{z}=Z\sin\alpha_{u},$$

(A5) $$v_{z}=Z\sin\alpha_{v},$$

(A6) $$w_{z}=Z\sin\alpha_{w}.$$

Following the same arguments for the derivation of Equations (4) and (5), we obtained

(A7) $$w_{h}=-H_{h}\cos\alpha_{w},$$

(A8) $$w_{x}=-X_{2}\cos\alpha_{w}=-X\cos\alpha_{w}\sin\left(\beta +\delta_{w}\right).$$

Following the same arguments for the derivation of the rest of the Galperin transformation matrix, we obtained

(A9) $$v_{x}=-X_{2}\cos\alpha_{v}=-X\cos\alpha_{v}\sin\left(\beta -\delta_{v}\right),$$

(A10) $$u_{x}=X\cos\alpha_{u},$$

(A11) $$u_{y}=0,$$

(A12) $$w_{y}=-Y_{2}\cos\alpha_{w}=-Y\cos\alpha_{w}\cos\left(\beta +\delta_{w}\right),$$

(A13) $$v_{y}=Y_{2}\cos\alpha_{y}=Y\cos\alpha_{y}\cos\left(\beta -\delta_{w}\right),$$

and

(A14) $$\left[\begin{array}{c} u\\ v\\ w \end{array}\right]=\left[\begin{array}{ccc} \cos\alpha_{u} & 0 & \sin\alpha_{u}\\ -\cos\alpha_{v}\sin\left(\beta -\delta_{v}\right) & \cos\alpha_{y}\cos\left(\beta -\delta_{w}\right) & \sin\alpha_{v}\\ -\cos\alpha_{w}\sin\left(\beta +\delta_{w}\right) & -\cos\alpha_{w}\cos\left(\beta +\delta_{w}\right) & \sin\alpha_{w} \end{array}\right]\left[\begin{array}{c} X\\ Y\\ Z \end{array}\right],$$

where $u=u_{x}+u_{y}+u_{z}$, $v=v_{x}+v_{y}+v_{z}$, and $w=w_{x}+w_{y}+w_{z}$. While the analytical expression for the inverse of this matrix can be easily found with an analytical tool such as Mathematica and MATLAB, it is more convenient in practice to perform the inversion numerically.

## Appendix C. Low-Frequency Signals with Arbitrary Angles and Un-Matched Seismometers

In this appendix, we present the most general form for the transformation matrix for signals of arbitrary frequencies with arbitrary angles and unmatched component seismometers.

(A15) $$\left[\begin{array}{c} u_{\omega }\\ v_{\omega }\\ w_{\omega } \end{array}\right]=-\omega^{2}\left[\begin{array}{ccc} \frac{\cos\alpha_{u}}{H_{ru}\left(\omega \right)} & 0 & \frac{\sin\alpha_{u}}{H_{ru}\left(\omega \right)}\\ -\frac{\cos\alpha_{v}\sin\left(\beta -\delta_{v}\right)}{H_{rv}\left(\omega \right)} & \frac{\cos\alpha_{y}\cos\left(\beta -\delta_{w}\right)}{H_{rv}\left(\omega \right)} & \frac{\sin\alpha_{v}}{H_{rv}\left(\omega \right)}\\ -\frac{\cos\alpha_{w}\sin\left(\beta +\delta_{w}\right)}{H_{rw}\left(\omega \right)} & -\frac{\cos\alpha_{w}\cos\left(\beta +\delta_{w}\right)}{H_{rw}\left(\omega \right)} & \frac{\sin\alpha_{w}}{H_{rw}\left(\omega \right)} \end{array}\right]\left[\begin{array}{c} X_{\omega }\\ Y_{\omega }\\ Z_{\omega } \end{array}\right],$$

where $H_{ru}\left(\omega \right)=\left(\omega_{ou}^{2}-\omega^{2}\right)+i\left(\omega_{su}^{2}\phi_{u}+\omega B_{u}/J_{u}\right)$, where the subscribe u denotes the quantities pertaining to the U component seismometer and is similarly defined for $H_{rv}\left(\omega \right)$ and $H_{rw}\left(\omega \right)$. Again, we recommend using numerical inversion for the matrix rather than the analytical form of the inverse matrix.

## Appendix D. Torque Feedback with Arbitrary Angles and Un-Matched Seismometers

In this appendix, we present the most general form for the transformation matrix for torque feedback operations with arbitrary angles and unmatched component seismometers.

(A16) $$\left[\begin{array}{c} \Gamma_{u}/\left(m_{u}D_{gu}\right)\\ \Gamma_{v}/\left(m_{v}D_{gv}\right)\\ \Gamma_{w}/\left(m_{w}D_{gw}\right) \end{array}\right]=\left[\begin{array}{ccc} \cos\alpha_{u} & 0 & \sin\alpha_{u}\\ -\cos\alpha_{v}\sin\left(\beta -\delta_{v}\right) & \cos\alpha_{y}\cos\left(\beta -\delta_{w}\right) & \sin\alpha_{v}\\ -\cos\alpha_{w}\sin\left(\beta +\delta_{w}\right) & -\cos\alpha_{w}\cos\left(\beta +\delta_{w}\right) & \sin\alpha_{w} \end{array}\right]\left[\begin{array}{c} \ddot{X}\\ \ddot{Y}\\ \ddot{Z} \end{array}\right],$$

where $m_{u}$, $m_{v}$ and $m_{w}$ are the total suspended masses for the U, V, and W component seismometers respectively, and $D_{gu}$, $D_{gv}$ and $D_{gw}$ are their center of masses respectively. Again, the inversion of this matrix is best carried out numerically.

## Appendix E. Time Domain Transformation in the Absence of Torque Feedback

When torque feedback is not used, this appendix shows how to process the data to obtain ground motion. The equation of motion (Equation (30)) can be rewritten as

(A17) $$\ddot{\theta }+\left[\frac{\omega_{s}^{2}\phi \left(\omega \right)}{\omega }+\frac{B}{J}\right]\dot{\theta }+\omega_{o}^{2}\theta =\left(\frac{mD_{g}}{J}\right)\left(\sin\alpha \ddot{Z}-\cos\alpha {\ddot{H}}_{h}\right).$$

The use of a frequency-dependent damping coefficient $\omega_{s}^{2}\phi \left(\omega \right)/\omega$ in the equation of motion [24] is equivalent to using an imaginary loss term. One should note that ϕ(ω) emphasizes that ϕ is also frequency dependent [25]. Applying this to the W component seismometer

(A18) $$\ddot{w}+\left[\frac{\omega_{sw}^{2}\phi_{w}\left(\omega \right)}{\omega }+\frac{B_{w}}{J_{w}}\right]\dot{w}+\omega_{ow}^{2}w=\left(\sin\alpha_{w}\ddot{Z}-\cos\alpha_{w}{\ddot{H}}_{h}\right).$$

Frequency-dependent damping implies that the measured w(t) must be expressed in its Fourier components before one can use Equation (A18). Since Fourier transform assumes that the signal being transformed is repetitive, w(t) must represent a time series that starts before a seismic event begins and ends after it has subsided. One can further simplify the calculation by choosing w(t) so that w=0 at the first and last points of the series. Let there be *N* data points in w(t). The Fourier representation of w(t) is

(A19) $$w\left(t\right)=\overset{N}{\underset{i=1}{\sum }}a_{wi}\sin\left(\omega_{i}t\right).$$

One can define $W\left(t\right)=\ddot{w}+\left[\frac{\omega_{sw}^{2}\phi_{w}\left(\omega \right)}{\omega }+\frac{B_{w}}{J_{w}}\right]\dot{w}+\omega_{ow}^{2}w$. Therefore, one can compute

(A20) $$W\left(t\right)=\ddot{w}+\left(\frac{B_{w}}{J_{w}}\right)\dot{w}+\omega_{ow}^{2}w+\omega_{sw}^{2}\overset{N}{\underset{i=1}{\sum }}a_{wi}\phi_{w}\left(\omega_{i}\right)\cos\left(\omega_{i}t\right).$$

One can similarly compute U(t) and V(t) from the measured values of u(t) and v(t).

Using the one-to-one mapping of equations, one can obtain the following transformation

(A21) $$\left[\begin{array}{c} U\\ V\\ W \end{array}\right]=\left[\begin{array}{ccc} \cos\alpha_{u} & 0 & \sin\alpha_{u}\\ -\cos\alpha_{v}\sin\left(\beta -\delta_{v}\right) & \cos\alpha_{y}\cos\left(\beta -\delta_{w}\right) & \sin\alpha_{v}\\ -\cos\alpha_{w}\sin\left(\beta +\delta_{w}\right) & -\cos\alpha_{w}\cos\left(\beta +\delta_{w}\right) & \sin\alpha_{w} \end{array}\right]\left[\begin{array}{c} \ddot{X}\\ \ddot{Y}\\ \ddot{Z} \end{array}\right].$$

The inversion of this should be conducted numerically.

One can see that without torque feedback, the process to recover ground acceleration is numerically intensive. This is especially true when the seismometer operates in a vacuum, where the internal friction in the spring is the dominant loss mechanism. On the Moon, the recovery process is even more intensive due to the long duration of seismic events [26].

## Appendix F. An Alternative Way to Derive the Brownian Noise as a Way to Illustrate the Importance of the Concept of the Null Point

The Brownian torque noise given by Equation (48) is $\left|\Gamma_{B\_\omega }\right|$. The angular acceleration Brownian noise of a single rotational mass-spring oscillator is therefore $\left|{\ddot{\theta }}_{B\_\omega }\right|=\left|\Gamma_{B\_\omega }\right|/J$, where J is the moment of inertia.

As discussed in Section 4, when the ground moves at a frequency higher than the resonance frequency, to an observer on an inertial frame, the location on the boom at the Null Point does not move. Only the ground moves up and down at an amplitude of Z. However, to an observer on the ground, the ground does not move, the Null Point moves up and down by the same displacement Z. Therefore, $\theta =Z\sin\alpha /D_{n},$ as given by Equation (25), can be used to compute the output Brownian noise of a seismometer as $\left|{\ddot{Z}}_{B\_\omega }\right|=\frac{D_{n}\left|\Gamma_{B\_\omega }\right|}{J\sin\alpha }$. Using our results of $D_{n}=\frac{J}{mD_{g}}$ as given by Equation (33), we obtain $\left|{\ddot{Z}}_{B\_\omega }\right|=\frac{\left|\Gamma_{B\_\omega }\right|}{mD_{g}\sin\alpha }$, which is consistent with the result of Erwin et al. [26] as shown in Equation (47), except for the extra factor of $1/\sqrt{3}$ which accounts for the noise reduction due to the averaging of noise from three component seismometers in a Galperin triaxial seismometer. If the location of the displacement sensor (or any other locations) were used instead of the location of the Null point, it would lead to an inconsistent result. Therefore, the concept of the Null Point is crucial in making the physics of the rotational mass-spring oscillator consistent.
